# The Relationship Between Valence and Chills in Music: A Corpus Analysis

**DOI:** 10.1177/20416695211024680

**Published:** 2021-07-27

**Authors:** Rémi de Fleurian, Marcus T. Pearce

**Affiliations:** Cognitive Science Research Group, Queen Mary University of London, UK; Cognitive Science Research Group, Queen Mary University of London, UK; Center for Music in the Brain, Aarhus University & Royal Academy of Music, Aarhus, Denmark

**Keywords:** chills, music, valence, corpus analysis

## Abstract

Chills experienced in response to music listening have been linked to both happiness and sadness expressed by music. To investigate these conflicting effects of valence on chills, we conducted a computational analysis on a corpus of 988 tracks previously reported to elicit chills, by comparing them with a control set of tracks matched by artist, duration, and popularity. We analysed track-level audio features obtained with the Spotify Web API across the two sets of tracks, resulting in confirmatory findings that tracks which cause chills were sadder than matched tracks and exploratory findings that they were also slower, less intense, and more instrumental than matched tracks on average. We also found that the audio characteristics of chills tracks were related to the direction and magnitude of the difference in valence between the two sets of tracks. We discuss these results in light of the current literature on valence and chills in music, provide a new interpretation in terms of personality correlates of musical preference, and review the advantages and limitations of our computational approach.

Chills are a pleasurable psychophysiological response, often accompanied by piloerection, which can occur in response to music listening. They have been of great interest in scientific research on music for their involvement in emotional and aesthetic responses to music, as suggested by phenomenological reports of the experience and empirical findings about their physiological and neural correlates (for a review, see de Fleurian & Pearce, 2020). Particular attention has been given to their elicitors, resulting in the identification of a range of acoustic and musical features usually associated with the experience of chills, such as sudden dynamic changes, increased roughness, crescendi, or the entrance of new instruments (see de Fleurian & Pearce, 2020). These features represent local musical and auditory events and are therefore reflective of the fleeting nature of chills.

In contrast to continuous changes within musical stimuli, emotional characteristics of entire musical pieces have also been investigated. As a result, chills have been associated with perceived valence. However, there is disagreement about the direction of this relationship. While Grewe et al. (2011) reported an increase in frequency of chills for positively valenced music, Panksepp (1995) found that chills were more frequently associated with perceived sadness. In the latter study, however, both happy and sad music were found to elicit chills, reflecting subsequent findings that both emotions are linked with chills when they are expressed by music (Bannister, 2020; Mori & Iwanaga, 2017; Panksepp, 1995).

Conflicting effects of valence on chills have been discussed in the context of being moved, a mixed emotional state involving sadness and joy (Menninghaus et al., 2015). More specifically, being moved has been associated with chills when listening to music (Bannister, 2019, 2020; Bannister & Eerola, 2018; Benedek & Kaernbach, 2011) and has been found to mediate the relationship between liking and sadness in response to music (Vuoskoski & Eerola, 2017). Moving stimuli often feature narrative displays of social separation or reunion (Wassiliwizky et al., 2015), prosocial behaviour (Wassiliwizky et al., 2017), or self-sacrifice (Konečni et al., 2007), but it remains unclear how such narrative features translate to music, and how stimulus valence relates to the occurrence of chills. It could be that sad music provides an emotional context more conducive to the occurrence of chills (Panksepp, 1995). Another plausible explanation for the mixed effects of valence on chills comes from the possibility that chills encompass several phenomenologically distinct experiences, partly characterised by different degrees of felt emotions (Bannister, 2019; Maruskin et al., 2012).

This perspective is further developed in a recent review (de Fleurian & Pearce, 2020), in which a preliminary model suggests three different pathways for the experience of chills, linking different types of elicitors to the combination of psychological and evolutionary mechanisms most likely to elicit chills, if not different types of chills. In one such pathway, individuals with high trait empathy are suggested to be more receptive to emotional elicitors of chills, such as perceived valence, leading them to mimic the perceived emotion through a process called emotional contagion, and to then experience chills through the process of being moved. Other pathways link acoustic and musical elicitors to processes involving arousal and musical expectation, respectively. There is little research on the existence of these pathways, and confirming or refuting an effect of perceived valence on chills would be a useful step in providing support for the existence of one of them, leading to a better understanding of the causes of chills and, in turn, of music appreciation in general.

In light of the current evidence, it remains difficult to establish the role of expressed stimulus valence on the incidence of chills in music. While behavioural approaches have contributed to identifying conflicting effects of happiness and sadness, they remain limited due to the number of stimuli which can be reasonably presented to participants, ranging here from 3 (Bannister & Eerola, 2018) to 23 (Grewe et al., 2011). Computational methods, however, can overcome such restrictions and are well suited to the study of a large collection of naturalistic stimuli, at the cost of reduced control over experimental conditions. There has been, to our knowledge, no use of corpus-based analysis in research on chills, despite the success of similar approaches in research on music and emotion (e.g., Eerola, 2011). The analysis presented in this article is an attempt at addressing this gap in the literature, focusing on the effects of valence and other track-level audio features (i.e., features computed over entire musical pieces) on chills in music.

Specifically, we compare features between tracks known to elicit chills and a control set of tracks matched by artist, duration, and popularity. First, we conduct confirmatory analyses regarding the effect of the valence feature on chills, hypothesising a difference in expressed valence between the two sets of tracks. Then, we conduct two exploratory analyses, to investigate the influence of other features on the occurrence of chills, and to assess whether these features influence the direction and magnitude of the difference in valence between both sets of tracks. We discuss these results and the advantages and limitations of our approach and provide a new interpretation with reference to a theory of the personality correlates of musical preference (Rentfrow et al., 2011), identifying relationships between chills and several dimensions capturing musical preference (*sophisticated* music and *intense* music).

## Materials

### Data Set

For this study, we used *ChiM* (version 1.0.0, as included in de Fleurian & Pearce, 2020), a data set prepared by compiling every mention of a piece of music reported to elicit chills in the literature reviewed in de Fleurian and Pearce (2020), whether these mentions consisted of author anecdotes, participant reports, empirical verifications, or discussion of prior results. This corresponds to 988 mentions of music confirmed to induce chills in at least one listener.

### Features

Track-level audio features were collected using *spotifyr* (Thompson et al., 2020), an R package that enables pulling track information from Spotify’s Web API (Spotify, n.d.). This allowed us to obtain, for most tracks, a range of features of interest, including *duration* (in milliseconds) and *popularity* (based on number and recency of plays), as well as nine track-level audio features: *acousticness* (confidence that the track is acoustic); *danceability* (based on tempo, rhythm stability, beat strength, and overall regularity); *energy* (based on dynamic range, perceived loudness, timbre, onset rate, and general entropy); *instrumentalness* (confidence that the track contains no vocal content); *liveness* (likelihood that the track was performed live); *loudness* (overall loudness in decibels); *speechiness* (presence of spoken words); *valence* (conveyed musical positiveness); and *tempo* (estimated in beats per minute). While Spotify does not share details about how these audio features are computed, they have successfully been used in previous research (e.g., Mas-Herrero et al., 2018; Melchiorre & Schedl, 2020).

## Methods

### Chills Tracks

We removed 136 duplicated tracks from ChiM, before looking up track information by sending API queries for strings containing the artist (or arranger/interpreter, as indicated in ChiM) and title of each track. The top result for each query was retained and used to pull the features described in the Features section. Throughout this process, an additional 103 tracks were removed due to unavailability on Spotify or missing audio features, resulting in a data set of 749 tracks which can cause chills.

### Matched Tracks

Our analysis aims to identify if specific track-level features are related to the occurrence of chills in ChiM. Therefore, a control set of tracks that do not cause chills was needed. Because it is impossible to assert that a specific track never causes chills for anyone, we approximated the construction of this control set. More specifically, we compared features across the chills tracks with features in another set of tracks, matched as fairly as possible with the chills tracks by artist, duration, and popularity. This strict matching procedure ensured that there were as few differences as possible between both sets of tracks other than their potential to elicit chills. While it is possible that some matched tracks can elicit chills as well (see the Discussion section for some limitations in our approach), it is unlikely that all of them do, meaning that any difference detected between the two sets of tracks can shed light on which factors affect the occurrence of chills.

First, we gathered potential matches by getting the first 50 tracks for each of the first 50 albums returned by an API query for each artist represented in the chills tracks. Then, we removed from these potential matches any track that was already present in the chills tracks, by comparing Spotify track IDs across the two sets. However, many duplicates remained across the two sets, as a piece of music on Spotify can have several distinct track IDs or slightly different titles. To mitigate this possibility, for each artist, we also removed from the potential matches any track with a title that had any number or any word of four letters or more (except the words *major* and *minor*) in common with the chills tracks. This process resulted in a pool of 205,717 potential matches.

**Figure 1.**
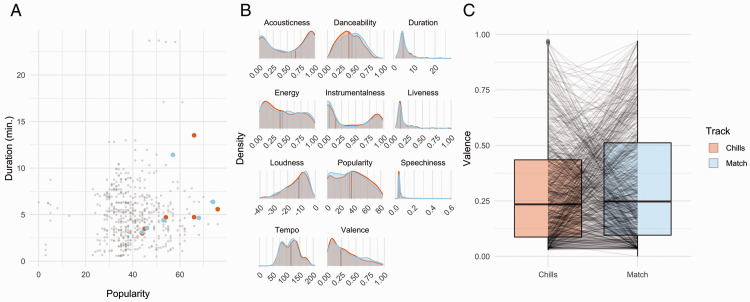
A: Example of the matching procedure, using Pink Floyd tracks. Tracks from ChiM are shown in orange, and potential matches gathered with the Spotify Web API are shown in grey. Potential matches with the shortest Euclidean distance from each chills track, in terms of duration and popularity, were selected as matched tracks, shown in blue. B: Densities and median values of audio features and metadata for the 722 resulting pairs of chills and matched tracks. C: Boxplots showing valence for the 722 pairs of tracks, with lines linking valence scores for each individual pair.

Finally, we standardised track duration and popularity across the chills tracks and the potential matches. For each artist, the potential matches with the shortest Euclidean distance from each chills track for these two standardised features were selected as the best matches (see Figure 1A). Audio features were pulled for these 749 matched tracks but were missing for 10 of them. A further 17 matched tracks were considered as outliers and removed for having Euclidean distances larger than three standard deviations from the mean, resulting in 722 pairs of chills and matched tracks (see Figure 1B and also Dataset S1 in the supplemental material for a full list of chills and matched tracks).

### Analysis

The confirmatory analysis consisted of assessing whether there is a difference in valence between chills tracks and matched tracks. We ran a logistic regression for the effect of the valence feature on track type (chills vs. matched). The presence of influential data points was checked with leave-one-out diagnostics, with the plan to run another logistic regression excluding data points that would, if left out, affect the slope by at least half of its original absolute value.

The exploratory analyses were twofold. First, we assessed whether there was a difference between chills tracks and matched tracks in the nine audio features described in the Features section. Due to high collinearity between these features, we first ran a principal component analysis (PCA), a method that projects each data point into a new dimensional space, the axes of which are called principal components, and allows for dimensionality reduction when retaining the first few components, which account for as much variance in the data as possible. PCA was run with centring and scaling, resulting in two principal components with eigenvalues above one—a common threshold to decide which components to retain based on how much variance they explain in the original data. We ran a logistic regression for the effects of these two principal components on track type (chills vs. matched), checking influential data points with leave-one-out diagnostics as described earlier.

Second, we assessed whether the audio features of the chills tracks had an effect on the *difference* in valence between chills tracks and matched tracks, which could mean that different types of chills arise in response to different auditory characteristics. As described earlier, we first ran a PCA due to collinearity, before running a linear regression for the effects of the two resulting principal components on the difference in valence between the chills tracks and the matched tracks, using leave-one-out diagnostics, and checking homoscedasticity and normality of residuals with residuals plots.

Finally, to check the robustness of the matching procedure, we conducted Wilcoxon signed-rank tests to compare duration and popularity between chills tracks and matched tracks, expecting no differences in duration and popularity. Nonparametric tests were chosen due to the fact that duration and popularity do not follow a normal distribution (see Figure 1B). Because there were some differences between both sets of tracks (see the Results section), we conducted mediation analyses, using the nonparametric bootstrap with 5000 Monte Carlo draws, as implemented in the *mediation* package (Tingley et al., 2014), to check if potential effects of the valence feature on track type were mediated by track duration and popularity. In addition, to mitigate this weakness of the matching procedure, all the analyses described earlier were replicated a total of 10 times, using a different set of matched tracks, each comprising one of the 10 tracks with the shortest Euclidean distance from each chills track (i.e., shortest Euclidean distance for iteration #1, second shortest for iteration #2, etc.)

## Results

### Effect of Valence on Track Type

A logistic regression model yielded a significant fit (χ^2^(1) = 6.33, *p* = .012, Nagelkerke $R^{2}=.006$ ), revealing a significant effect of the valence feature on track type (*b* = 0.54, *Z* = 2.51, *p* = .012), with the valence of chills tracks being lower than that of matched tracks by 0.033 on a 0–1 scale (see Figure 1C). This effect remained significant across all 10 iterations of the analysis (mean valence difference =0.042 , *SD* = 0.009). Results for the 10 iterations are shown in Table S1.

### Mediating Effects of Duration and Popularity

A Wilcoxon signed-rank test revealed no significant difference in duration (*V* = 137199, *p* =.232) and a significant difference in popularity (*V* = 134593, *p* =.003) between chills tracks and matched tracks (higher for chills tracks by 2.90 on a 1–100 scale), suggesting that the matching procedure did not result in an optimal set of matched tracks. The difference in popularity remained significant in all 10 iterations of the analysis, while the difference in duration became significant in the fourth as well as the last five iterations of the analysis, presumably due to the increasing Euclidean distance between chills tracks and each successive set of matched tracks (see Table S2).

To assess whether duration and popularity mediated the effect of the valence feature on track type as reported in the previous section, we conducted two separate causal mediation analyses. For duration, the average causal mediation effect was not significant ( ACME=−.006 , *p* = .716), and the average direct effect was significant ( ADE=−.128 , *p* =.021), suggesting that duration did not mediate the effect of valence on track type. For popularity, both average effects were significant (*ACME* =.037, *p* =.002; ADE=−.171 , *p* <.001), suggesting that popularity partially, but not fully, mediated the effect of valence on track type. These results remained stable across the 10 iterations of the analysis, except for duration, which partially mediated the effect of valence on track type in the last iteration (see Table S3).

In this case, the mediation analyses each involved a linear regression (for the effect of valence on duration/popularity) and a logistic regression (for the effects of valence and duration/popularity on track type). It is worth noting that for the linear models, some assumptions (homoscedasticity and normality of residuals) were violated. This is most likely due to the distribution of valence, duration, and popularity in our data (see Figure 1B). To confirm the results of the mediation analyses, we ran them again on a reduced (keeping only tracks with nonzero popularity due to this feature being zero-inflated) and transformed data set (square root for popularity and log for valence and duration). These reanalyses did not fully eliminate the violations of assumptions for linear regression but did replicate the findings presented earlier (see Table S4).

### Effects of Audio Features on Track Type

We ran a PCA to reduce collinearity in the nine audio features. We retained two principal components with eigenvalues higher than one, accounting for 56.4% of cumulative proportion of variance explained (see Dataset S1 for the values for each set of track). The first component featured high positive loadings (greater than .2) for energy, loudness, valence, danceability, and tempo, and high negative loadings (lower than –.2) for acousticness and instrumentalness. The second component featured high positive loadings for liveness and speechiness, and a high negative loading for danceability. The number of retained principal components and their associated loadings were consistent across all 10 iterations of the analysis (besides occasional but systematic sign differences, which are expected when conducting several PCAs—see Table S5).

**Figure 2.**
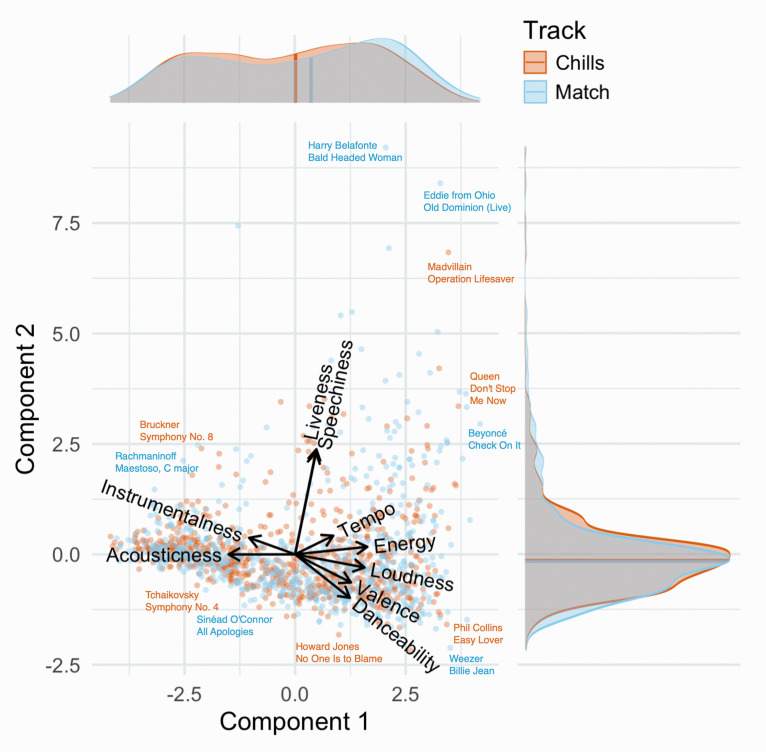
Biplot of chills and matched tracks for the first iteration of the analysis. Tracks are mapped onto the first two components obtained with PCA. Some example tracks are shown for various combinations of component values. Densities and median values for chills and matched tracks are shown in marginal plots, revealing a difference on Component 1. Audio feature loadings are shown as vectors, illustrating the high degree of collinearity between some features.

A logistic regression model yielded a significant fit (χ^2^(2) = 6.47, *p* = .039, Nagelkerke $R^{2}=.006$ ), revealing a significant effect of the first component to assess if properties of the on track type (*b* = 0.06, *Z* = 2.34, *p* =.019) and no significant effect of the second component (*b* = 0.05, *Z* = 0.98, *p* =.328), showing that chills tracks had lower scores than matched tracks on the first component (i.e., chills tracks featured lower energy, loudness, valence, danceability, and tempo, as well as higher acousticness and instrumentalness—see Figure 2). The model fit remained significant in all but one iteration of the analysis (χ^2^(2) = 5.33, *p* = .070, Nagelkerke $R^{2}=.005$ ), the effect of the first component remained significant in all iterations, and the effect of the second component became significant in four iterations, highlighting that in some cases, chills tracks had lower scores than matched tracks on the second component (i.e., low liveness and speechiness, as well as high danceability). It is worth noting that in the seventh iteration of the analysis, there were two influential data points (as described in the Analysis section). For this iteration, we ran the model both with and without the influential data points, leading to similar results in both cases (see Table S6).

### Effects of Audio Features on Difference in Valence Between Track Types

We ran a PCA on the nine audio features of the chills tracks only (as opposed to both track types in the previous section), to assess if properties of the chills track can predict the direction and magnitude of the difference in the valence feature between both track types. We retained two principal components with eigenvalues higher than one, accounting for 55.6% of cumulative proportion of variance explained. Both components featured similar loadings as in the previous section. The number of retained principal components and their associated loadings were consistent across all 10 iterations of the analysis (see Table S7).

A multiple linear regression model yielded a significant fit, F(2,719)=63.9 , *p* < .001, adjusted $R^{2}=.149$ , revealing a significant effect for both the first component ( β=.062 , *p* <.001) and the second component ( β=.039 , *p* <.001), suggesting that chills tracks with higher scores on these components were more likely to be happier than their associated matched tracks, and vice versa. These effects held for all 10 iterations of the analysis (see Table S8).

## Discussion

### Results

In this experiment, we compared track-level audio features between tracks taken from ChiM, a data set of pieces of music known to elicit chills, and several sets of tracks algorithmically matched by artist, duration, and popularity.

We compared the valence feature between chills tracks and matched tracks and found that chills tracks were, on average, slightly lower in valence. This echoes previous findings that chills are more frequently associated with perceived sadness (Panksepp, 1995), as opposed to perceived happiness (Grewe et al., 2011). The matching procedure resulted in a small difference in valence between track types, but it is worth noting that overall, the distribution of valence in ChiM is highly positively skewed, whereas it is relatively uniform across all tracks on Spotify (Spotify, n.d.). In other words, an effect of valence was identified despite the application of a strict matching procedure, which most likely resulted in high similarity between chills tracks and matched tracks. If control tracks had been selected randomly instead, most chills tracks would have had a much lower valence by comparison.

When taking all audio features into consideration, we found that chills tracks were characterised by smaller values on a component linked with high energy, loudness, valence, danceability, and tempo, as well as low acousticness and instrumentalness, meaning that overall, chills track were sadder, slower, less intense, and more instrumental than matched tracks. In a few occasions, chills tracks were also characterised by smaller values on a second component linked with high liveness and speechiness, as well as low danceability, therefore suggesting that chills-inducing music may be less likely to include spoken words and to feature a live audience, although these results are less robust than those for the first component. These findings can be interpreted with reference to an influential theory of the personality correlates of musical preference (Rentfrow et al., 2011). The musical characteristics we identified strongly match *sophisticated* music, which tends to be relaxing, quiet, nondanceable, slow, nonelectric, and instrumental (Rentfrow et al., 2012), suggesting that chills tracks were more sophisticated than matched tracks in our analysis. Interestingly, preference for sophisticated music is associated with openness to experience (Schäfer & Mehlhorn, 2017), a personality characteristic strongly linked to the experience of chills (see de Fleurian & Pearce, 2020).

We also examined whether the audio features of chills tracks relate to the difference in valence between chills tracks and matched tracks. We found that tracks with higher energy, loudness, valence, liveness, and speechiness, as well as lower acousticness and instrumentalness, were more likely to be happier than their associated matched tracks, and vice versa. While these results are partly explained by valence loading on the first component obtained with PCA, the involvement of other audio features suggests a potential interpretation. Using the same classification as earlier (Rentfrow et al., 2012), it becomes apparent that, on average, *sophisticated* chills tracks were sadder than matched tracks, and *intense* chills tracks (i.e., nonrelaxing, loud, electric, and featuring raspy or yelling voice) were happier than matched tracks. As is the case with sophisticated music, intense music is linked with openness to experience (Schäfer & Mehlhorn, 2017), a known personality correlate of chills. Interestingly, these results provide some support for the possibility that different types of chills are elicited by different types of feelings and affective states expressed or evoked by music (Bannister, 2019; Maruskin et al., 2012), and for the possible presence of several pathways for the experience of chills (de Fleurian & Pearce, 2020).

### Limitations

We discussed the rationale and some limitations of the matching procedure in the Matched Tracks section. Notably, despite our best efforts, matched tracks were slightly less popular than chills tracks. This could be due to a bias towards popular tracks when reporting music that causes chills. It should be possible to reduce this difference by picking matched tracks from a larger set of potential matches, but our current methods did not allow this due to limits on the rate of Spotify API requests and the number of records per request. As a result, we found that popularity partially, but not fully, mediated the difference in valence between chills tracks and matched tracks. This could be interpreted as sad songs being more popular and, in turn, popular songs being more likely to cause chills. However, the mediation analyses were not part of the planned primary analysis, but rather only intended as a procedural check, and we suspect the identified mediating effects are largely due to the idiosyncrasies of our data. Nonetheless, we attempted to address any limitations in the matching procedure by conducting 10 iterations of the full analysis with different sets of matched tracks, which led to consistent results across iterations.

There are other limitations to our approach. ChiM does not report the exact version of the pieces of music that elicit chills, which could have had some impact on the audio characteristics of the chills tracks. Then, some chills tracks might have been present in the matched set, despite our efforts to limit this possibility (see the Matched Tracks section). In general, apart from a few sanity checks, we considered the lack of manual verification of our data as an acceptable trade-off for the large size of the data set, which made a robust computational analysis possible. Another issue is the lack of transparency about how Spotify computes audio features. Again, we accepted this trade-off that allowed us to collect large amounts of audio data and metadata through API queries.

Importantly, effect sizes were small for most of our results. One possible explanation is that we tried to be as fair as possible with the matching procedure, which might have drastically reduced the differences in audio features between chills tracks and matched tracks—effect sizes would probably have been more pronounced if we randomly selected matched tracks, but this process would have introduced noise due to confounding differences between both track types. Also, as highlighted in the Matched Tracks section, it is possible that some matched tracks also had the ability to elicit chills. Finally, chills are a localised phenomenon, and it is fully expected that track-level features would not capture local changes in acoustic and structural features, therefore limiting the explanatory power of our approach. However, we believe that the consistency of the results across several iterations of the analysis on different sets of matched tracks yielded robust and interpretable findings, despite their small effect size.

### Conclusion

We conducted a corpus analysis of audio characteristics of music known to elicit chills and identified that such music is sadder than music matched by artist, duration, and popularity. Exploratory analyses revealed that chills tracks are also slower, less intense, and more instrumental than matched tracks on average and tend to possess the characteristics of sophisticated music. Moreover, within chills tracks, we identified a possible relationship between valence and type of music, with sophisticated music tending to be sadder than matched tracks, and intense music tending to be happier. These results show that, for research about chills in music, computational methods have a great and largely untapped potential to complement empirical studies.

## Supplemental Material

sj-zip-1-ipe-10.1177_20416695211024680 - Supplemental material for The Relationship Between Valence and Chills in Music: A Corpus Analysis Click here for additional data file. Supplemental material, sj-zip-1-ipe-10.1177_20416695211024680 for The Relationship Between Valence and Chills in Music: A Corpus Analysis by Rémi de Fleurian and Marcus T. Pearce in i-Perception

sj-pdf-2-ipe-10.1177_20416695211024680 - Supplemental material for The Relationship Between Valence and Chills in Music: A Corpus Analysis Click here for additional data file. Supplemental material, sj-pdf-2-ipe-10.1177_20416695211024680 for The Relationship Between Valence and Chills in Music: A Corpus Analysis by Rémi de Fleurian and Marcus T. Pearce in i-Perception
