# Supplementary material for: The Relationship Between Valence and Chills in Music: A Corpus Analysis
Source: Iperception. 2021 Jul 27;12(4):20416695211024680. doi: 10.1177/20416695211024680 (PMC8323431; doi:10.1177/20416695211024680)
Supplement: sj-pdf-2-ipe-10.1177_20416695211024680 - Supplemental material for The Relationship Between Valence and Chills in Music: A Corpus Analysis [file sj-pdf-2-ipe-10.1177_20416695211024680.pdf]

Supplementary tables

Table S1: Effect of valence on track type for all 10 iterations of the analysis.

	**Model fit**			**Valence**		
Iteration	χ^2	p	Nagelkerke R^2	b	Z	p
1	6.33	.012	.006	0.54	2.51	.012
2	12.04	$<$ .001	.011	0.75	3.45	$<$ .001
3	8.99	.003	.008	0.65	2.99	.003
4	5.13	.023	.005	0.51	2.26	.024
5	13.13	$<$ .001	.012	0.80	3.60	$<$ .001
6	6.42	.011	.006	0.56	2.53	.012
7	11.72	$<$ .001	.011	0.75	3.40	$<$ .001
8	15.46	$<$ .001	.014	0.83	3.91	$<$ .001
9	15.88	$<$ .001	.015	0.86	3.96	$<$ .001
10	14.98	$<$ .001	.014	0.84	3.84	$<$ .001

Table S2: Difference in duration and popularity between chills tracks and matched tracks.

	**Duration**		**Popularity**	
Iteration	V	p	V	p
1	137199	.232	134593	.003
2	142583	.051	145166	$< .001$
3	142185	.060	150597	$< .001$
4	146264	.005	152310	$< .001$
5	140323	.059	154629	$< .001$
6	141824	.026	159205	$< .001$
7	145974	.005	159076	$< .001$
8	144000	.013	162740	$< .001$
9	143062	.010	163894	$< .001$
10	144711	.004	165209	$< .001$

Table S3: Mediating effects of duration and popularity for the effect of valence on track type.

	Duration				Popularity			
Iteration	*ACME*	*p*	*ADE*	*p*	*ACME*	*p*	*ADE*	*p*
1	-.006	.716	-.128	.021	.037	.002	-.171	$< .001$
2	-.010	.503	-.173	.002	.049	$< .001$	-.232	$< .001$
3	-.012	.434	-.147	.004	.063	$< .001$	-.223	.001
4	-.028	.103	-.100	.095	.067	$< .001$	-.196	$< .001$
5	-.012	.419	-.184	$< .001$	.073	$< .001$	-.270	$< .001$
6	-.020	.232	-.118	.034	.072	$< .001$	-.209	$< .001$
7	-.019	.209	-.164	.002	.073	$< .001$	-.256	$< .001$
8	-.016	.267	-.189	$< .001$	.075	$< .001$	-.281	$< .001$
9	-.022	.145	-.188	.001	.073	$< .001$	-.285	$< .001$
10	-.032	.046	-.174	.002	.081	$< .001$	-.288	$< .001$

Table S4: Re-analyses of mediating effects of duration and popularity for the effect of valence on track type.

	**Duration**				**Popularity**			
Iteration	*ACME*	*p*	*ADE*	*p*	*ACME*	*p*	*ADE*	*p*
1	-.002	.656	-.023	.109	.010	.001	-.036	.010
2	-.005	.304	-.034	.024	.013	$< .001$	-.052	$< .001$
3	-.006	.136	-.019	.174	.017	$< .001$	-.043	.002
4	-.009	.086	-.024	.102	.020	$< .001$	-.052	$< .001$
5	-.006	.217	-.030	.032	.020	$< .001$	-.056	$< .001$
6	-.007	.094	-.017	.242	.021	$< .001$	-.045	$< .001$
7	-.008	.051	-.028	.058	.023	$< .001$	-.059	$< .001$
8	-.005	.198	-.028	.049	.023	$< .001$	-.056	$< .001$
9	-.008	.070	-.036	.014	.021	$< .001$	-.065	$< .001$
10	-.010	.026	-.036	.016	.026	$< .001$	-.072	$< .001$

Table S5: Principal component analysis on audio features for all tracks.

		**Audio feature loadings**								
PC	Iteration	Tempo	Loudness	Valence	Dance.	Energy	Acoust.	Instru.	Speech.	Liveness
1	1	.242	.439	.353	.346	.457	-.419	-.291	.132	.134
	2	.222	.444	.356	.346	.459	-.422	-.298	.128	.118
	3	.233	.438	.350	.343	.458	-.421	-.289	.151	.142
	4	.220	.447	.351	.341	.461	-.428	-.300	.112	.117
	5	.221	.442	.356	.343	.461	-.422	-.297	.139	.117
	6	.243	.439	.354	.338	.456	-.420	-.292	.153	.124
	7	.231	.439	.346	.341	.459	-.425	-.301	.151	.115
	8	.241	.437	.352	.348	.453	-.419	-.302	.135	.125
	9	.220	.441	.356	.348	.456	-.421	-.293	.151	.124
	10	.241	.438	.351	.351	.456	-.422	-.292	.130	.122
2	1	.119	-.083	-.178	-.275	.048	-.001	.105	.645	.665
	2	.158	.078	.080	.148	.003	-.071	.046	-.660	-.706
	3	.138	-.029	-.293	-.365	.081	-.051	.098	.548	.665
	4	.075	-.052	-.240	-.300	.062	-.055	.020	.581	.706
	5	.050	.019	.219	.324	-.063	.029	.013	-.567	-.719
	6	.007	-.020	-.261	-.359	.076	-.048	.007	.498	.739
	7	.043	-.010	-.276	-.363	.048	-.038	-.045	.543	.699
	8	.037	.078	.150	.202	.017	-.046	.056	-.644	-.714
	9	.052	-.075	-.173	-.267	.020	.012	.035	.612	.717
	10	.088	.100	.101	.162	.007	-.051	.033	-.654	-.717

Note. PC = Principal component, Dance. = Danceability, Acoust. = Acousticness, Instru. = Instrumentalness, Speech. = Speechiness.

Table S6: Effect of first two principal components on track type.

	Model fit			Component 1			Component 2		
Iteration	χ^2	p	Nagelkerke R^2	b	Z	p	b	Z	p
1	6.47	.039	.006	0.06	2.34	.019	0.05	0.98	.328
2	10.39	.006	.010	0.07	2.42	.016	-0.11	-2.10	.036
3	6.57	.038	.006	0.07	2.51	.012	0.03	0.51	.612
4	13.04	.001	.012	0.07	2.63	.008	0.12	2.44	.015
5	9.00	.011	.008	0.07	2.76	.006	-0.06	-1.17	.243
6	5.33	.070	.005	0.06	2.15	.032	0.04	0.83	.404
7a	6.71	.035	.006	0.07	2.56	.010	0.02	0.37	.714
7b	6.96	.031	.006	0.07	2.61	.009	0.02	0.40	.687
8	13.28	.001	.012	0.07	2.74	.006	-0.12	-2.37	.018
9	7.20	.027	.008	0.07	2.61	.009	0.03	0.60	.551
10	12.53	.002	.012	0.07	2.69	.007	-0.12	-2.26	.024

Note. The analysis for iteration 7 was conducted with (7a) and without (7b) influential data points.

Table S7: Principal component analysis on audio features for chills tracks only.

		**Audio feature loadings**								
PC	Iteration	Tempo	Loudness	Valence	Dance.	Energy	Acoust.	Instru.	Speech.	Liveness
1	1	.251	.440	.345	.339	.463	-.419	-.283	.155	.126
	2	.249	.440	.346	.336	.463	-.420	-.283	.158	.127
	3	.249	.440	.347	.335	.463	-.421	-.283	.157	.125
	4	.247	.441	.346	.334	.464	-.421	-.282	.153	.129
	5	.249	.440	.346	.337	.463	-.420	-.281	.156	.129
	6	.253	.439	.344	.337	.463	-.420	-.282	.157	.125
	7	.250	.440	.346	.335	.463	-.421	-.282	.158	.126
	8	.250	.440	.346	.335	.463	-.421	-.282	.158	.126
	9	.249	.439	.346	.334	.463	-.421	-.285	.159	.126
	10	.250	.438	.345	.338	.462	-.420	-.286	.155	.127
2	1	.014	.088	.149	.265	-.020	-.007	-.037	-.650	-.690
	2	.008	.083	.164	.283	-.030	.004	-.049	-.646	-.682
	3	.009	.081	.164	.281	-.029	.004	-.047	-.646	-.684
	4	.005	.084	.162	.284	-.030	.005	-.049	-.654	-.674
	5	.025	.086	.148	.273	-.024	-.005	-.040	-.648	-.688
	6	.016	.086	.157	.269	-.024	-.004	-.040	-.647	-.689
	7	.015	.088	.152	.274	-.025	-.001	-.047	-.646	-.688
	8	.015	.088	.152	.274	-.025	-.001	-.047	-.646	-.688
	9	.009	.084	.162	.277	-.028	.000	-.052	-.646	-.685
	10	.011	.082	.160	.277	-.031	.000	-.048	-.650	-.682

Note. PC = Principal component, Dance. = Danceability, Acoust. = Acousticness, Instru. = Instrumentalness, Speech. = Speechiness.

Table S8: Effect of first two principal components on difference in valence between track types.

	**Model fit**			**Component 1**		**Component 2**	
Iteration	F	p	Adjusted R^2	β	p	β	p
1	63.9	$< .001$	.149	0.062	$< .001$	0.039	$< .001$
2	62.5	$< .001$	.145	0.059	$< .001$	0.040	$< .001$
3	65.2	$< .001$	.151	0.063	$< .001$	0.033	.002
4	64.8	$< .001$	.150	0.059	$< .001$	0.031	.002
5	55.7	$< .001$	.132	0.057	$< .001$	0.028	.007
6	47.5	$< .001$	.115	0.052	$< .001$	0.026	.012
7	51.8	$< .001$	.124	0.055	$< .001$	0.033	.002
8	51.4	$< .001$	.123	0.057	$< .001$	0.035	.002
9	51.7	$< .001$	.124	0.058	$< .001$	0.033	.003
10	55.2	$< .001$	.131	0.058	$< .001$	0.035	.001
